# PredβTM: A Novel β-Transmembrane Region Prediction Algorithm

**DOI:** 10.1371/journal.pone.0145564

**Published:** 2015-12-22

**Authors:** Amrita Roy Choudhury, Marjana Novič

**Affiliations:** Laboratory of Chemometrics, National Institute of Chemistry, Ljubljana, Slovenia; Russian Academy of Sciences, Institute for Biological Instrumentation, RUSSIAN FEDERATION

## Abstract

Predicting the transmembrane regions is an important aspect of understanding the structures and architecture of different β-barrel membrane proteins. Despite significant efforts, currently available β-transmembrane region predictors are still limited in terms of prediction accuracy, especially in precision. Here, we describe PredβTM, a transmembrane region prediction algorithm for β-barrel proteins. Using amino acid pair frequency information in known β-transmembrane protein sequences, we have trained a support vector machine classifier to predict β-transmembrane segments. Position-specific amino acid preference data is incorporated in the final prediction. The predictor does not incorporate evolutionary profile information explicitly, but is based on sequence patterns generated implicitly by encoding the protein segments using amino acid adjacency matrix. With a benchmark set of 35 β-transmembrane proteins, PredβTM shows a sensitivity and precision of 83.71% and 72.98%, respectively. The segment overlap score is 82.19%. In comparison with other state-of-art methods, PredβTM provides a higher precision and segment overlap without compromising with sensitivity. Further, we applied PredβTM to analyze the β-barrel membrane proteins without defined transmembrane regions and the uncharacterized protein sequences in eight bacterial genomes and predict possible β-transmembrane proteins. PredβTM can be freely accessed on the web at http://transpred.ki.si/.

## Introduction

Integral membrane proteins are divided into two distinct classes based on their structural motifs–the α-helical membrane proteins and the β-barrel membrane proteins. The β-transmembrane (βTM) proteins are embedded in the outer membranes of gram-negative bacteria, mitochondria, chloroplasts, and cell wall of gram-positive bacteria [1]. They exhibit a diverse range of functional categories that include porin, transporter, adhesin, lipase, protease, deacylase, pore-forming toxin, and assembly factors [2,3]. Their crucial roles, particularly in the influx and efflux of small molecules, in bacteria make the βTM proteins important candidates for targeting and delivery of antimicrobial drugs, as well as for understanding multidrug resistance.

The βTM proteins are difficult to identify in the genome sequences and hence their exact number is unknown. A rough estimate suggests that they account for no less than a few percentages of the open reading frames [4]. However, the structures of very few non-homologous βTM proteins have been determined. They amount to only ~0.28% of the PDB structures [5,6], with 162 protein structures representing the 29 known β-barrel transmembrane protein superfamilies [3].

All the structurally resolved βTM proteins are closed barrels of short antiparallel β-strands ensuring maximum neighborhood correlation [1]. The basic structural subunit of transmembrane β-barrels is the β-hairpin. Regardless of the similar structural features, the βTM proteins show diversity in architecture and topology [7].

Although a well-defined set of rules can describe the structurally resolved transmembrane β-barrels [1], to date, the prediction of the transmembrane regions and structure of the βTM proteins remains a difficult problem. A major factor contributing to this limitation is the sparse sampling, and it is likely that there are βTM protein families that are yet to be described.

Identifying the transmembrane regions is the first step towards the structural elucidation of a βTM protein. Several methods to predict the β-transmembrane regions have been developed based on Neural Networks (NN), Support Vector Machines (SVM) and Hidden Markov Models (HMM), and a few of the developed algorithms are available freely [8–12]. Since the amphipathic nature of the short transmembrane β-strands does not allow the efficient use of hydrophobicity as a discriminating factor, most of the complex algorithms incorporate non-linear statistics and evolutionary profiles. Yet, the performance of the algorithms is limited.

In this work, we present the PredβTM algorithm for transmembrane β-strand prediction. It utilizes the amino acid pair frequency information of a given protein sequence. Additional information about explicitly defined evolutionary profiles is not considered in the prediction model. This information is implicitly incorporated as statistical preferences resulting from encoding the proteins into amino acid adjacency matrix. Further, PredβTM is also independent of hydrophobicity index. The algorithm has a two-layered architecture with an underlying support vector machine (SVM) [13] classifier. When tested with a benchmark set of 35 βTM proteins, PredβTM is able to predict the β-transmembrane strands with a sensitivity of 83.71% and precision of 72.98%. The predicted transmembrane regions show a segment overlap of 82.19%. While maintaining a sensitivity that is as high as the best available predictors, PredβTM shows significant improvement in terms of prediction precision, as well as has a higher segment overlap score. Finally, we have applied the developed algorithm to analyze and predict the probable transmembrane regions for the known βTM proteins and uncharacterized proteins in selected bacterial genomes.

## Materials and Methods

### βTM protein data

βTM protein sequences and transmembrane region information are collected from public domain databases PDB and PDBTM [5,6]. After the initial data refinement, a dataset of 101 βTM protein sequences, with experimentally determined structures and transmembrane region annotations, are obtained. These sequences are then separated into their transmembrane and non-transmembrane counterparts. Only the part of the β-strand that is embedded in the membrane, and not the whole β-strand that may extend beyond it, is considered as a transmembrane region. The long non-transmembrane regions are further segmented into ~10 residues long fragments, such that the transmembrane and non-transmembrane regions are of comparable lengths. Thus we obtain 3632 segments in total; of which 1423 are transmembrane and the remaining 2209 are non-transmembrane. Each segment is labeled accordingly. This main dataset is divided into two, which are then used to train and validate the SVM classifier that forms the core of the PredβTM algorithm.

### Mathematical encoding of the segments

**Fig 1:**
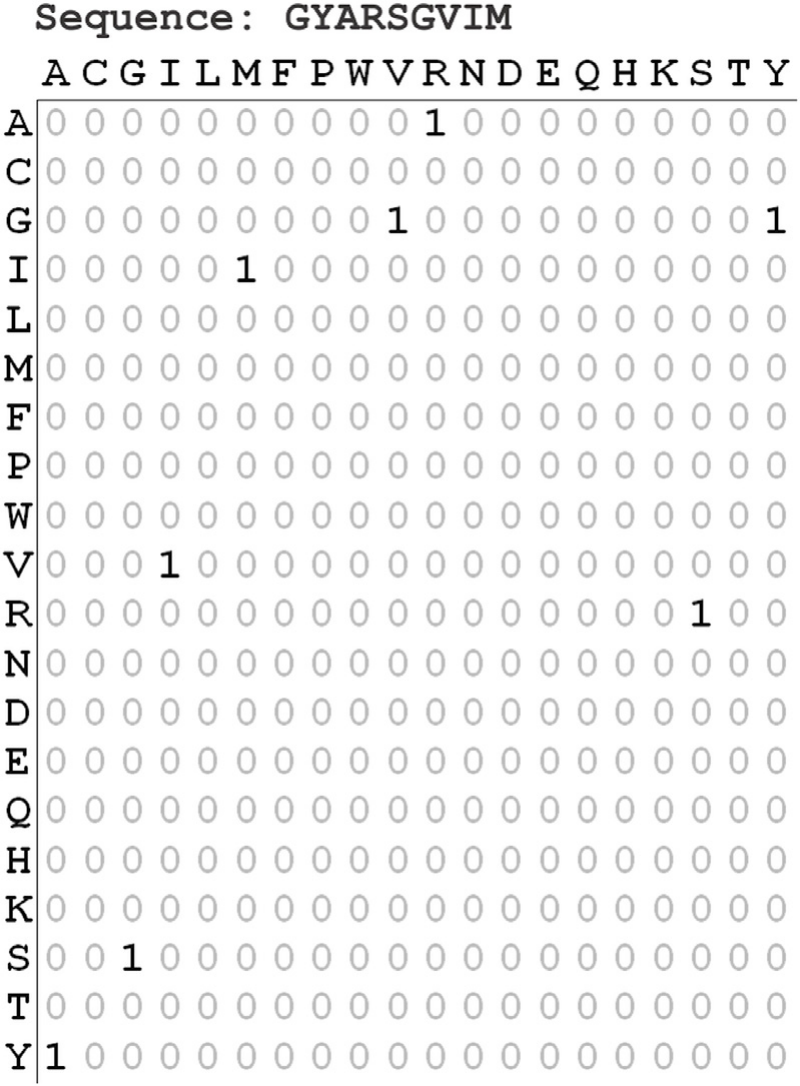
Amino acid adjacency matrix. The 20×20 amino acid adjacency matrix of the given βTM protein segment is shown. The matrix elements representing the frequency of the corresponding amino acid pairs in the segment are highlighted.

The amino acid adjacency matrix [14] is used to mathematically encode the protein segments into their amino acid pair frequency. It is a 20×20 matrix with the rows and columns labeled with the 20 natural amino acids (Fig 1). Each element of the matrix represents the frequency of the corresponding amino acid pair in the given sequence. The descriptor vector or the mathematical representation of a given protein segment is generated considering all 400 matrix elements.

Other matrix invariants, like rowsum, do not show significant changes in performances for previously built classifiers using Counter-Propagation Neural Network (CPNN). Using Support Vector Machine (SVM), on the other hand, shows diminished performance for classifier developed with matrix invariant rowsum. The rowsum essentially presents the frequency of amino acids in a given peptide sequence. Data condensation performed to calculate the rowsum results in loss of the amino acid adjacency information that inherently encodes the sequence profiles. As this information is crucial for the classifier performance, the 400-dimensional descriptor vector is chosen. The resulting descriptor vectors are sparse in nature due to the short length of the protein segments considered and encode the amino acid pair frequency information.

### SVM classifier

The first layer of the developed PredβTM algorithm is a two-class classifier built using Support Vector Machines (SVM) [13] implemented with LIBSVM [15].

To train and validate the SVM classifier, the original dataset is divided into two mutually exclusive training and validation sets. In generating the training and validation sets, there is always a trade-off problem; (i) keeping all information from the data set to make the prediction model as sound as possible; and (ii) loosing information from the dataset by assigning certain amount of information to be excluded in order to be used for the validation of the model. Sparse sampling is a major contributing factor to the limited performance of β-transmembrane region predictors. Therefore, in order to retain the complete representation and variability of the available data space in the training set, the training and validation sets are not generated at the protein subunit level. It is also important to ensure that the profiles inherently generated using amino acid adjacency matrix capture the diverse information present in the βTM protein data.

The training and validation sets are generated using Kohonnen Neural Networks (KNN) [16]. Kohonnen maps are self-organized topological feature maps that attempt to preserve the topology of the input information while mapping onto the neural array such that the topologically similar protein segments are grouped together on the map. Both training and the validation sets consist of at least one sample from each non-empty neuron on the map. This ensures that both sets contain comprehensive representations of the available data space. The number of protein segments in the training and validation sets is 3044 and 588, respectively. The ratio of non-transmembrane to transmembrane segments in each dataset is ~1.5.

For building the βTM classifier, we have chosen the non-linear Radial Basis Function (RBF) kernel, which is capable of efficient handling of non-linear relationships between the class labels and the descriptors. Using linear kernel shows diminished classifier performance. As the relationship between the numerical characterization of the protein segments and their classification as transmembrane or non-transmembrane is non-linear, RBF kernel proves to be a better choice in building the classifier. The performance of the RBF kernel depends on two parameters–a) soft-margin constant *C* that assigns penalty to misclassification and margin errors, and b) kernel parameter *γ* that determines the decision boundary. Only the training set is used for the parameter optimization and βTM classifier development. In the final step, the developed classifier is challenged with the validation set to assess the classifier performance.

### PredβTM algorithm

The PredβTM algorithm is developed to predict the β-strand transmembrane regions of a given protein sequence. The algorithm has a two-layered architecture. In the first layer, the developed βTM classifier is used to make the initial classification of transmembrane and non-transmembrane segments. The second layer incorporates statistical data on position-specific amino acid preference patterns [17] to fine-tune and make the final transmembrane region predictions. The algorithm is implemented with ActivePerl 5.16.1. The implementation of PredβTM is summarized below, where the notation ‘:’ defines a range (See S1 Fig for the flowchart).

1. A given protein sequence is segmented into overlapping segments, each 10 residues long. A protein with *n* residues, therefore, yields *n-9* segments *S1*, *S2*, *…*., *S{n-9}*.
2. Each of these segments is mathematically encoded to obtain its numerical representation (400-dimensional descriptor vector).
3. The βTM SVM classifier predicts whether each of the *n-9* segments is either transmembrane (*tm*) or non-transmembrane (*ntm*).
4. If the segments *Si*, *S{i+1}*, *S{i+2}*, *…*, *S{i+j}* are predicted as *tm* such that *j ≥ 4*, and the segments are consecutive, then the residues covered (*R = Si*_*1*_:*S{i+j}*_*10*_, where *Sx*_*y*_ represents the *y* ^*th*^ residue of the segment *Sx*) by such consecutive overlapping segments predicted as *tm* are considered for the final prediction.
5. *R* is further sub-segmented into *Rseg = {r1*, *r2*, *…*, *rn}* such that *r1 = Si*_*1*_:*Si*_*10*_, *r2 = Si*_*1*_:*S{i+1}*_*10*_, *…*, *rn = S{i+j}*_*1*_:*S{i+j}*_*10*_, and *6 ≤ length(r(i)) ≤ 12*. The termini of these segments are then scored according to position-specific amino acid preference data.
6. One of the top three positive scoring segments that shows maximum overlap with the central residues of the region *R* is finally reported as the transmembrane region TM. *Top3 = {r(i) | r(i)∈ Rseg for all i = 1*, *2*, *3*, *and r(i) > r(j) for all j = 4*:*n*, *r(j) ∈ Rseg}*. *TM = {r(i) | mid(r(i)) → mid(R) and r(i) ∈ Top3}*.

### Benchmark data

A benchmark dataset is used to evaluate the performance of the developed PredβTM algorithm and compare it with other state-of-art β-transmembrane region predictors. It contains 35 βTM protein sequences with experimentally known transmembrane regions obtained from the TOPDB database [18] (See S1 Table). While training and validation sets only contain βTM proteins with known structural information, the benchmark set contains proteins with experimentally annotated β-transmembrane regions. The βTM proteins considered in the benchmark dataset are not used in any other steps of algorithm development.

## Results

First we present the βTM SVM classifier, which forms the core of the algorithm. Then we discuss in detail the working of the developed β-transmembrane region predictor PredβTM with an example. Lastly, we report the performance of PredβTM on the benchmark set, and discuss it in comparison with other available algorithms. We also applied the algorithm to analyze the uncharacterized outer membrane proteins in eight bacterial genomes.

### βTM SVM classifier

The βTM classifier forms the first layer and the core of the PredβTM algorithm, imparting a significant influence on the algorithm performance. Therefore, parameter optimization is a crucial step in building the SVM classifier. Exhaustive parameter search and optimization is achieved by performing a two-level grid-search with 10-fold cross-validation using the training set. The cross-validation procedure prevents the problem of over-fitting or over-training. The optimized (*C*,*γ*) values are (7.4642, 0.125) with a cross-validation accuracy rate of 92.77%.

Our training set contains unbalanced data, i.e. the non-transmembrane to transmembrane sample ratio is 1.56. Therefore, a high-accuracy classifier will essentially be the majority-class classifier, i.e. the non-transmembrane class classifier in this case, and may lead to higher number of false negative predictions. To correct this disparity in data, two separate soft-margin constants (*C*_*TM*_, *C*_*nTM*_) are used for the transmembrane and non-transmembrane classes, and are set to values (7.4624, 4.7905). This assures that the misclassification penalty assigned to each class is reflective of its sample size.

The βTM classifier is then trained with the training dataset using the optimized parameters *C*_*TM*_, *C*_*nTM*_ and *γ*. The self-consistency test, i.e. assessing the trained classifier with the training set itself, shows a recall ability of 99.97% (3043 of 3044 samples are classified correctly).

The prediction accuracy of the trained βTM classifier is also estimated with the validation set, which is not used in any step of parameter optimization and classifier building. The estimated prediction accuracy is 94.22%, with 34 of the 588 samples in validation set being misclassified. The number of misclassified samples here refers to the transmembrane and non-transmembrane segments, and not whole proteins. It must be noted that the validation set only estimates the performance of the SVM classifier, which forms the first layer of the algorithm. It is therefore only reflective of the algorithm performance and does not give the actual estimation of PredβTM performance.

### PredβTM–transmembrane β-strand predictor

Given a protein sequence, the PredβTM algorithm predicts the transmembrane β-strands present in the protein. The initial prediction is performed by the βTM SVM classifier. It is then refined using position-specific amino acid preference data [17] to generate the final transmembrane region prediction. The detail of the PredβTM algorithm is explained with the example of Outer membrane protein A (PDB ID 1QJP) [19]. It is a 171 residues long protein with eight experimentally determined transmembrane β-strands.

The first step of the PredβTM algorithm is to segment the input protein sequence into short overlapping segments of 10 residues. The window size of 10 is selected based on the membrane thickness and the lengths of the known transmembrane β strands. The 171 *(n)* residues long protein, in this example, generates 162 segments *(n-9)*. Each segment is 10 residues long, and has nine residues common with its immediate adjacent segments. Each segment is then mathematically represented with its amino acid pair frequency (400-dimensional descriptor vector), which is calculated using the amino acid adjacency matrix.

**Fig 2:**
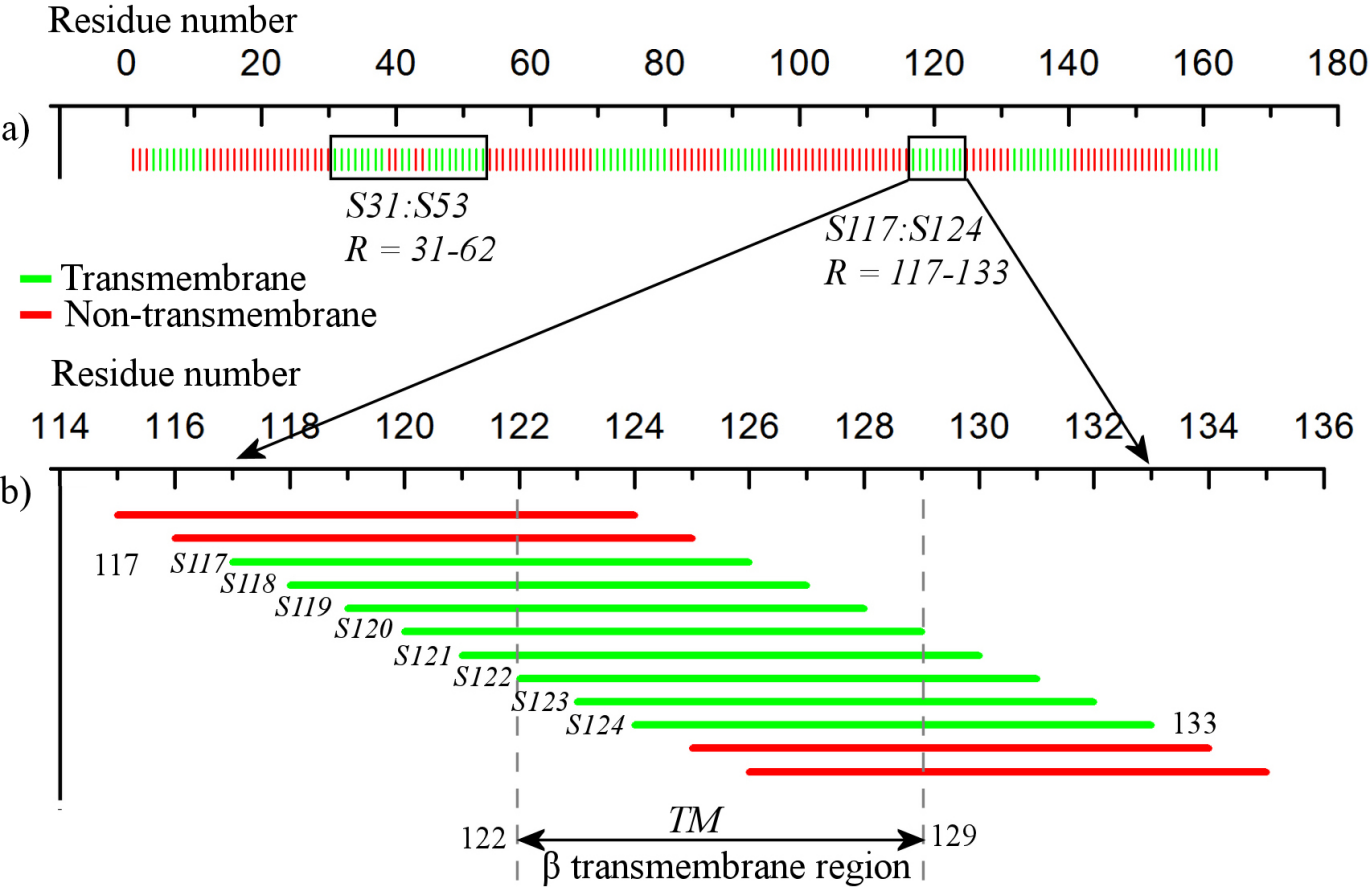
Prediction of transmembrane regions by the PredβTM algorithm. a) The vertical colored bars represent the first residues of the 162 segments of the protein 1QJP. The segments in green are predicted as *tm*, and those in red as *ntm* by the βTM SVM classifier. Two stretches of consecutive *tm* segments are highlighted (*S31*:*S53* and *S117*:*S124*). (b) The *tm* stretch *R* covering residues 117–133 is enlarged. The constituent segments (*S117*:*S124*) are shown in their full lengths to illustrate the overlap. The reported transmembrane region (*TM* = 122–129) is highlighted.

The βTM classifier then predicts each of these 162 segments as either transmembrane *(tm)* or non-transmembrane *(ntm)*. Fig 2A presents the prediction from βTM classifier, where each bar denotes the position of the first residue of the corresponding protein segment. The green bars indicate the segments classified as *tm*, while the red bars represent those classified as *ntm*. This classification forms the first layer of the PredβTM algorithm.

In the next step, the algorithm searches for four or more consecutive segments that are predicted as *tm*. Such consecutive *tm* segments span over a minimum of 13 residues, which allows enough room for considering segment overlaps and predicting statistically plausible transmembrane region boundary residues. All the residues contained by the consecutive *tm* segments are considered for refinement and final transmembrane region prediction. In our example, seven such stretches of consecutive *tm* segments are identified (visible as green patches in Fig 2A): 4:11 (8 segments), 31:53 (23 segments), 70:80 (11 segments), 89:96 (8 segments), 117:124 (8 segments), 132:140 (9 segments), and 156:162 (7 segments). Intervening isolated *ntm* segments are allowed if the stretch extends beyond six *tm* segments. The algorithm is further explained with the example of the stretch of residues *R* = 117–133 *(S117*_*1*_:*S124*_*10*_ *)* corresponding to the eight consecutive *tm* segments *S117*:*S124*.

Fig 2B presents the transmembrane stretch *R* in detail. As segments predicted as *tm* overlap with *ntm* segments, only the central residues of the stretch *R* are considered as the core residues of the predicted transmembrane region. To decide the transmembrane region boundary residues and refine the initial prediction by the βTM SVM classifier, the algorithm uses the position-specific amino acid preference data. This part forms the second layer of the algorithm.

For this purpose, all possible combinations of the constituent *tm* segment termini are considered to form the sub-segments of *R*, *Rseg = {r1*, *r2*, *…*, *r43}*, such that the length of each sub-segment is between 6 and 12 residues. The boundary residues of each segment are scored using the position-specific amino acid preference data obtained by statistical analysis of structurally resolved βTM protein sequences [17]. Positive score is awarded for the statistically preferred amino acids at the terminal positions; otherwise, there is a penalty of negative score. The elements of *Rseg* are ranked according to their scores.

In the final step, the three highest positive-scoring sub-segments *(Top3)* in *Rseg* are considered. The top scoring sub-segment that shows maximum overlap with the central residues of the transmembrane stretch *R* is reported as the final β-transmembrane region (*TM*). In this example, the final β-transmembrane region *TM* is predicted at residues 122–129 (Fig 2B). This predicted region shows maximal overlap with the central residues of *R* (122–128) and exactly matches the experimentally predicted transmembrane region. It must be noted that the position-specific amino acid preference data affects only the selection of the transmembrane region boundary residues, and is incorporated only to predict more statistically favourable β-transmembrane regions.

The β-hairpins form the structural unit of transmembrane β-barrels, often resulting in very short non-transmembrane loops of 2–5 residues formed by the tight turns. The sliding window scheme of the PredβTM algorithm makes the distinction of such short non-transmembrane loops difficult. In such cases, the βTM classifier reports a long stretch of consecutive *tm* segments, often marked by isolated *ntm* segments. This long stretch encompasses both the β-strands of the β-hairpin. In our example, the stretch *S31*:*S53* presents one such β-hairpin. In cases like this, *R* is divided into non-overlapping sub-regions *(R*_*1*_, *R*_*2*_, *…*, *R*_*n*_ *)*, where each sub-region is approximately 13 residues long and is treated independently. In our example *R* = 31–62 *(S31*_*1*_:*S53*_*10*_ *)* can be divided into two sub-regions. Following the previously explained methodology, each sub-region is separately analyzed to predict the two transmembrane regions at residues 35–44 and 49–61. The predicted regions correspond to the experimental β-transmembrane regions at residues 37–44 and 49–55, respectively.

### Benchmarking PredβTM

The performance of PredβTM algorithm is estimated with the benchmark dataset of 35 βTM proteins. A true positive prediction is defined when a predicted transmembrane region overlaps with exactly one of the experimentally observed transmembrane regions. It must be noted that the reported accuracy for most algorithms is overestimated and reflects the recall ability of the method rather than its ability to interpolate or extrapolate. As the number of well annotated βTM proteins is limited, the benchmark set may include proteins that are close homologs of proteins in the original dataset of PredβTM training, as well as in the training sets of other methods compared. It is impossible to avoid the presence of remote homologs in the benchmark set without compromising with maintaining maximum variability in the training set, which is important for algorithm performance. This, however, does not imply that the benchmarking method is biased. Instead of calculating absolute values of the measures of accuracy, it is only possible to measure the upper limit of the prediction accuracy for the algorithms due to the fact that all the βTM protein families are likely not yet identified or well annotated. Some of the known βTM protein families do not have any structural representation. Since only proteins with structural data were considered for training the Pred βTM algorithm, while the benchmark set exclusively contains proteins without structural information, the possibility of presence of close homologs is low. Moreover, as PredβTM training set contains protein fragments instead of complete protein sequences, possible presence of close homologs will result only in short local similarity instead of global alignments.

With the given benchmark dataset, PredβTM shows a sensitivity of 83.71% and a positive predictive value or precision of 72.27%. The segment overlap is calculated to be 82.19%. The algorithm correctly predicts 370 of the 442 transmembrane regions present in the 35 benchmark sequences. Additionally, PredβTM also predicts 70 false positive transmembrane β-strands, and fails to predict 72 β-transmembrane regions. The transmembrane regions for only two of the benchmark sequences (BP00456 and BP01002) are predicted with 100% accuracy. The detail of the prediction for each of the benchmark sequences is presented in S2 Table.

βTM proteins are also present in eukaryotes, most of which are not well annotated. The algorithm is challenged to predict the transmembrane regions of mouse VDAC1 protein (voltage-dependent anion-selective channel protein 1), which is a mitochondrial outer membrane protein. It consists of 20 annotated β-transmembrane regions, 15 of which are correctly predicted by PredβTM. Although the algorithm fails to identify five of the transmembrane regions, it does not give any false positive prediction.

The performance of PredβTM is compared with five other freely available β-transmembrane region predictors: B2TMPRED [8], TBBpred [9], ConBBPRED [10], TMBETA-NET [11], and TMBpro [12]. B2TMPRED is feed-forward neural network based predictor that is trained with a non-redundant set of β-barrel membrane proteins [8]. It uses HSSP derived sequence profiles an input for the network to improve prediction accuracy. TMBpro also uses a neural network based predictor, where the sequence profiles in non-redundant database aligned to the target sequence serve as the input to the network [12]. Algorithm TMBETA-NET, which also uses a neural network model, is based on the statistical analysis of amino acid composition in well-annotated outer membrane proteins and globular proteins [11]. TBBpred is a combination of two different models–neural network based and SVM based [9]. The neural network based model incorporates evolutionary information as a multiple sequence alignment obtained from PSI-BLAST. The SVM model on the other hand uses primary sequence input along with physiochemical parameters of amino acids. The consensus method ConBBPRED [10] gives a prediction based on output from other available algorithms including PRED-TMBB, ProfTMB, BETA-TM, PSI-PRED etc.

It is not possible to compare the performances of individual methods, as they appear in the original publications, with that of PredβTM. This is because different methods use diverse input datasets and different annotations of transmembrane regions while reporting their performances. Hence for a reliable comparison, all the five predictors are used to predict the transmembrane regions of the 35 βTM proteins in the benchmark set. Table 1 presents the comparative performances of PredβTM and other available β-transmembrane region predictors.

**Table 1:** Comparative performance analysis of the developed PredβTM algorithm and five other available algorithms.

Algorithm	Known TM β-strands	Predicted TM β-strands	True positives	%Sensitivity^a^	%Precision^b^	%Segment Overlap
**PredβTM**	**442**	**512**	**370**	**83.71**	**72.58**	**82.26**
B2TMpred	442	872	367	83.03	42.09	73.16
TBBpred	442	807	328	74.21	40.64	79.57
ConBBPred	442	288	249	56.33	86.46	70.75
TMBETA-NET	442	690	317	71.72	45.94	65.05
TMBpro	442	471	330	74.66	70.06	73.31

^a^Sensitivity: TP/(TP+FN), % of all observed transmembrane β-strands predicted correctly by the model.

^b^Precision (Positive predictive value): TP/(TP+FP), % of all predicted transmembrane β-strands that are correctly predicted.

The developed predictor PredβTM outperforms all the available β-transmembrane region predictors, considered in this work, in terms of sensitivity, precision and segment overlap. Although the consensus method ConBBPRED [10] shows a considerably higher precision (86.46%), this high precision is achieved at the cost of lower sensitivity (56.55%). ConBBPRED radically fails to identify transmembrane β-strands leading to highest false negative prediction among the algorithms considered in this analysis. The evolutionary information based predictor B2TMPRED [8] shows sensitivity (83.03%) similar to that of PredβTM, however, the precision of the algorithm is much lower (42.09%), as well is the segment overlap (73.16%). Among the available predictors analyzed here, TMBpro shows the overall best performance with the sensitivity and precision of 74.66% and 70.06%, respectively.

Except TMBETA-NET, the other four algorithms considered for benchmarking explicitly use evolutionary information in the form of sequence profiles. Although incorporation of such information has shown to significantly improve performances for secondary structure and α-transmembrane region predictors, their influence is not yet clear for βTM proteins [10]. TMBETA-NET, which is based on amino acid composition, performs comparably with TBBpred and TMBpro, two methods that incorporate sequence profile information. PredβTM differs from other methods compared here in the fact that it does not explicitly incorporate sequence profiles and physicochemical properties of amino acids. The sequence profile information is captured automatically as statistical preferences in the amino acid pair frequency information. This is also the primary reason to incorporate the complete representation of the available data space in the training set. Additionally, the scoring based on position-specific amino acid preference information improves the prediction for transmembrane region boundaries, thus improving the segment overlap score. PredβTM uses a sliding window approach, where a given protein sequence is fragmented into overlapping segments, which are then classified. However, a transmembrane region is identified based on the consensus prediction for each constituent segment of the region. Therefore, isolated segments predicted as transmembrane due to the error rate within the classifier do not contribute to false positive predictions, increasing the precision of PredβTM algorithm. Considering adjacent overlapping segments also takes into account the presence of underlying correlation, like local compositional bias, among the transmembrane and non-transmembrane regions.

### Application

We have applied the developed PredβTM algorithm to analyze the known outer membrane proteins, as well as the uncharacterized proteins, of selected bacterial genomes. For this analysis, we consider eight gram-negative bacterial genomes that include two strains of *E*. *coli* (the virulent strain O157:H7 and cultivated strain K12) [20] and six other pathogenic bacterial genomes (strains mentioned in brackets): *S*. *aeruginosa* (PA01) [21], *Y*. *pestis* (CO92) [22], *H*. *influenza* (RdKW20) [22], *S*. *typhi* (CT18) [22], *S*. *dysenteriae* (Sd197) [23], *H*. *pylori* (26695) [24]. The annotated protein products of these eight genome assemblies are downloaded from NCBI Genomes ftp site. PredβTM is used to predict the probable transmembrane β-strands for the proteins annotated as outer-membrane, as well as for the uncharacterized hypothetical proteins.

The prediction results for the known outer-membrane proteins are presented in Table 2. The eight bacterial genomes contain 259 known βTM proteins. The predictor identifies 258 of these proteins as βTM with at least one predicted β-transmembrane region. For 79 of these proteins, there is some experimental information regarding their transmembrane β-strands. PredβTM predicts the β-transmembrane regions for these 79 proteins with 91.22% sensitivity and 88.71% precision. Transmembrane regions are also predicted for the remaining 180 outer-membrane proteins, for which the β-transmembrane regions are not yet identified experimentally.

**Table 2:** Transmembrane β-strand prediction for the proteins annotated as outer-membrane in eight bacterial genomes.

Strains	No. of proteins	βTM proteins		βTM proteins with available TM data						βTM proteins with no TM data
		Total	Predicted	Total	TM regions	Predicted TM regions	True positives	%Sensitivity^a^	%Precision^b^	
O157:H7 (*E*. *coli*)	5285	49	49	17	202	203	180	89.11	88.67	32
K12 (*E*. *coli*)	4140	62	61	23	306	302	280	91.50	92.72	39
PA01 (*S*. *aeruginosa*)	5571	29	29	6	98	87	80	81.63	91.95	23
CO92 (*Y*. *pestis*)	3797	29	29	10	104	126	104	100	82.54	19
RdKW20 (*H*. *influenza*)	1609	7	7	4	15	36	15	100	41.67	3
CT18 (*S*. *typhi*)	3141	40	40	8	112	103	97	86.61	94.17	32
Sd197 (*S*. *dysenteriae*)	4062	30	30	11	154	162	148	96.10	91.36	19
26695 (*H*. *pylori*)	1593	13	13	0	NA	NA	NA	NA	NA	13
**TOTAL**	**29198**	**259**	**258**	**79**	**991**	**1019**	**904**	**91.22**	**88.71**	**180**

The outer-membrane proteins with known transmembrane β-strands are analyzed in detail to obtain the prediction sensitivity and precision.

^a^Sensitivity: TP/(TP+FN), % of all observed transmembrane β-strands predicted correctly by the model.

^b^Precision: TP/(TP+FP), % of all predicted transmembrane β-strands that are correctly predicted.

Although PredβTM is not developed to analyze whole genomes and to distinguish between transmembrane and globular proteins, nevertheless, it is used to analyze the uncharacterized proteins in the genomes (Table 3). 1046 of the 7972 uncharacterized proteins are predicted as βTM with at least one predicted β-transmembrane region (Table 3). This serves only as an indication, and not as the true estimation, of the βTM proteins present among the uncharacterized proteins in the bacterial genomes. PredβTM does not predict any of the uncharacterized proteins in *E*. *coli* (K12) as potential βTM. Only 1.5% of the genome is identified as outer-membrane proteins. The estimated β-barrel proteins in *S*. *aeruginosa* and *H*. *pylori*, on the other hand, are 7.79% and 6.60% of the genomes, respectively. However, this can be an over estimation, arising due to false positive predictions. For *H*. *influenza*, one of the smallest gram-negative genomes, 55 βTM proteins (3.41% of the genome) are identified. In general, analysis with PredβTM indicates that around 2–5% of the gram-negative bacterial genomes code for outer-membrane β-barrel proteins.

**Table 3:** Predicted and known βTM proteins in eight bacterial genomes.

Strains	No. of proteins	Uncharacterized proteins		Known βTM proteins	%Genome
		Total	Predicted as βTM		
O157:H7 (*E*. *coli*)	5285	1854	182	49	4.37
K12 (*E*. *coli*)	4140	21	0	62	1.51
PA01 (*S*. *aeruginosa*)	5571	2308	405	29	7.79
CO92 (*Y*. *pestis*)	3797	1064	142	29	4.50
RdKW20 (*H*. *influenza*)	1609	389	48	7	3.41
CT18 (*S*. *typhi*)	3141	889	100	40	4.54
Sd197 (*S*. *dysenteriae*)	4062	832	81	30	2.73
26695 (*H*. *pylori*)	1593	615	88	13	6.60
TOTAL	29198	7972	1046	259	4.47

Uncharacterized proteins in the genomes that are predicted as βTM proteins by PredβTM are listed. The percentages of the genomes that are identified as βTM proteins (known and predicted by PredβTM) are also mentioned.

## Conclusion

We describe PredβTM, a novel algorithm to predict the transmembrane β-strands in membrane proteins. The training data contains a complete representation of the available βTM protein space with known structural information. The algorithm is based on amino acid pair frequency and is independent of explicitly defined evolutionary profile information. The algorithm implicitly captures this information as statistical preferences resulting from encoding the proteins into amino acid adjacency matrix. Position-specific amino acid preference data is used to refine the prediction by fine-tuning the transmembrane region boundaries. The algorithm shows a high sensitivity of 83.71%, and outperforms other available βTM predictors. Considering the complete data space while training and implicit incorporation of evolutionary information works better than other algorithms with pre-calculated alignment based sequence profiles. The algorithm is also used to analyze selected gram-negative bacterial genomes in order to predict the transmembrane regions in unannotated βTM proteins and give an indication of the transmembrane β-barrels present in the genomes. PredβTM can be accessed freely via a web interface.

## Supporting Information

S1 Fig The PredβTM algorithm. (TIFF)

S1 Table Benchmark dataset of 35 βTM proteins. (DOCX)

S2 Table Transmembrane region prediction results for the 35 βTM proteins from the benchmark dataset using the PredβTM algorithm. (DOCX)

## References

[1] Schulz G. β-Barrel membrane proteins. Curr. Opin. Struct. Biol. 2000; 10: 443–447. 10981633 10.1016/s0959-440x(00)00120-2

[2] Koebnik R, Locher KP, van Gelder P. Structure and function of bacterial outer membrane proteins: barrels in a nutshell. Mol. Microbiol. 2002; 37: 239–253. 10.1046/j.1365-2958.2000.01983.x 10931321

[3] Lomize MA, Lomize AL, Pogozheva ID, Mosberg HI. OPM: Orientations of Proteins in Membranes database. Bioinformatics 2006; 22: 623–625. 16397007 10.1093/bioinformatics/btk023

[4] Wimley WC. Towards genomic identification of β-barrel membrane proteins: Composition and architecture of known structures. Protein Sci. 2002; 11: 301–312. 11790840 10.1110/ps.29402 PMC2373429

[5] Berman HM, Westbrook J, Feng Z, Gilliland G, Bhat TN, Weissig H, et al The Protein Data Bank. Nucl. Acids Res. 2000; 28: 235–242. 10592235 10.1093/nar/28.1.235 PMC102472

[6] Tusnády GE, Dosztányi Z, Simon I. Transmembrane proteins in the protein data bank: Identification and classification. Bioinformatics 2004; 20: 2964–2972. 15180935 10.1093/bioinformatics/bth340

[7] Wimley WC. The versatile β-barrel membrane protein. Curr. Opin. Struct. Biol. 2003; 13: 404–411. 12948769 10.1016/s0959-440x(03)00099-x

[8] Jacoboni I, Martelli PL, Fariselli P, De Pinto V, Casadio R. Prediction of the transmembrane regions of β-barrel membrane proteins with a neural network-based predictor. Protein Sci. 2001; 10: 779–787. 11274469 10.1110/ps.37201 PMC2373968

[9] Natt NK, Saini H, Raghava GP. Prediction of transmembrane regions of β-barrel proteins using ANN- and SVM-based methods. Proteins: Struct. Funct. Bioinf. 2004; 56: 11–18. 10.1002/prot.20092 15162482

[10] Bagos PG, Liakopoulos TD, Hamodrakas SJ. Evaluation of methods for predicting the topology of beta-barrel outer membrane proteins and a consensus prediction method. BMC Bioinformatics 2005; 6: 7–19. 15647112 10.1186/1471-2105-6-7 PMC545999

[11] Gromiha MM, Ahmad S, Suwa M. TMBETA-NET: discrimination and prediction of membrane spanning β-strands in outer membrane proteins. Nucl. Acids Res. 2005; 33: W164–W167. 15980447 10.1093/nar/gki367 PMC1160128

[12] Randall A, Cheng J, Sweredoski M, Baldi P. TMBpro: secondary structure, β-contact and tertiary structure prediction of transmembrane β-barrel proteins. Bioinformatics 2008; 24: 513–520. 18006547 10.1093/bioinformatics/btm548

[13] Cortes C, Vapnik V. Support-vector network. Machine Learning 1995; 20: 273–297.

[14] Randić M, Novič M, Vračko M. On novel representation of proteins based on amino acid adjacency matrix. SAR QSAR Environ. Res. 2008; 19: 339–349. 10.1080/10629360802085082 18484502

[15] Chang CC, Lin CJ. LIBSVM: a library for support vector machines. ACM TISI 2011; 2: 27:2–27:27.

[16] Zupan J, Novič M, Ruisanchez I. Kohonen and counter-propagation artificial neural networks in analytical chemistry. Chemom. Int. Lab. Syst. 1997; 38: 1–23.

[17] Roy Choudhury A, Novič M. Amino acid distribution in transmembrane regions: a statistical analysis and comparison with globular proteins. Int. J. Chem. Model. 2012; 4: 205–219.

[18] Tusnády GE, Kalmár L, Simon I. TOPDB: topology databank of transmembrane proteins, Nucl. Acids Res. 2008; 36: D234–D239. 17921502 10.1093/nar/gkm751 PMC2238857

[19] Pautsch A, Schulz GE. High-resolution structure of the OmpA membrane domain. J. Mol. Biol. 2000; 298: 273–282. 10764596 10.1006/jmbi.2000.3671

[20] Zhang W, Nadrik J, Kossow A, Bielaszewska M, Leopold SR, Witten A, et al Phylogeny and phenotypes of clinical and environmental Shiga toxin-producing Escherichia coli O174. Environ. Microbiol. 2014; 16: 963–976. 10.1111/1462-2920.12234 24034719

[21] Stewart L, Ford A, Sangal V, Jeukens J, Boyle B, Kukavica-Ibrulj I, et al Draft genomes of 12 host-adapted and environmental isolates of Pseudomonas aeruginosa and their positions in the core genome phylogeny. Pathog. Dis. 2014; 71: 20–25. 10.1111/2049-632X.12107 24167005

[22] Wright MS. New insights into dissemination and variation of the health care-associated pathogen Acinetobacter baumannii from genomic analysis. MBio. 2014; 5: e00963–13. 10.1128/mBio.00963-13 24449752 PMC3903280

[23] Kaur G, Sathyabama S, Arora A, Verma S, Mubin N, Agrewala JN, et al Genome sequencing, annotation and comparative genomic analysis of Shigella dysenteriae strain SD1D. Gut Pathog. 2014; 6: 28 10.1186/1757-4749-6-28 25028600 PMC4099087

[24] Lu W, Wise MJ, Tay CY, Windsor HM, Marshall BJ, Peacock C, et al Comparative analysis of the full genome of Helicobacter pylori isolate Sahul64 identifies genes of high divergence. J. Bacteriol. 2014; 196: 1073–83. 10.1128/JB.01021-13 24375107 PMC3957704

