# Supplementary figures and images for: PredβTM: A Novel β-Transmembrane Region Prediction Algorithm
Source: PLoS One. 2015 Dec 22;10(12):e0145564. doi: 10.1371/journal.pone.0145564 (PMC4687927; doi:10.1371/journal.pone.0145564)

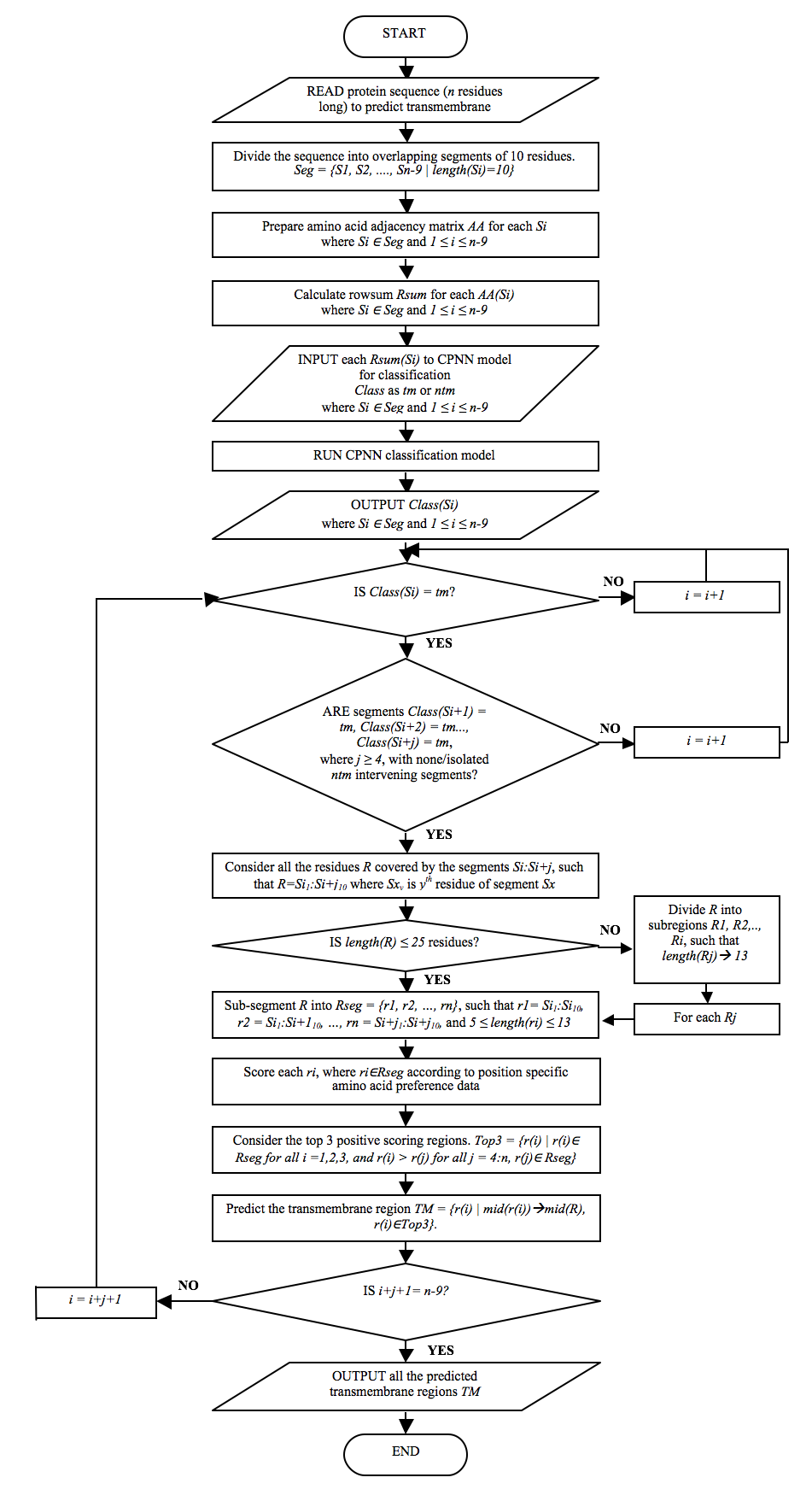

Supplement: S1 Fig — (TIFF) [file pone.0145564.s001.tiff]
