# Supplementary material for: PredβTM: A Novel β-Transmembrane Region Prediction Algorithm
Source: PLoS One. 2015 Dec 22;10(12):e0145564. doi: 10.1371/journal.pone.0145564 (PMC4687927; doi:10.1371/journal.pone.0145564)
Supplement: S2 Table — (DOCX) [file pone.0145564.s003.docx]

# Supporting Information

**S2 Table. Transmembrane region prediction results for the 35 βTM proteins from the benchmark dataset using the PredβTM algorithm.**

| ID (TOPDB) | Known TM β-strand | Predicted TM β-strand | True positives | %Sensitivity | %Precision |
| --- | --- | --- | --- | --- | --- |
| BP00115 | 8 | 9 | 8 | 100 | 88.89 |
| BP00310 | 18 | 20 | 18 | 100 | 90 |
| BP00320 | 2 | 3 | 2 | 100 | 66.67 |
| BP00359 | 16 | 19 | 14 | 87.5 | 73.68 |
| BP00374 | 22 | 20 | 18 | 81.82 | 90 |
| BP00376 | 12 | 9 | 9 | 75 | 100 |
| BP00382 | 10 | 9 | 8 | 80 | 88.89 |
| BP00384 | 16 | 12 | 11 | 68.75 | 91.67 |
| BP00398 | 10 | 11 | 10 | 100 | 90.91 |
| BP00402 | 22 | 27 | 22 | 100 | 81.48 |
| BP00421 | 8 | 10 | 8 | 100 | 80 |
| BP00424 | 18 | 17 | 16 | 88.89 | 94.12 |
| BP00451 | 8 | 6 | 6 | 75 | 100 |
| BP00456 | 8 | 8 | 8 | 100 | 100 |
| BP00463 | 16 | 10 | 9 | 56.25 | 90 |
| BP00496 | 14 | 15 | 14 | 100 | 93.33 |
| BP00497 | 12 | 11 | 10 | 83.33 | 90.91 |
| BP00504 | 22 | 29 | 14 | 63.64 | 48.28 |
| BP00505 | 12 | 36 | 12 | 100 | 33.33 |
| BP00506 | 2 | 14 | 1 | 50 | 7.14 |
| BP00517 | 4 | 11 | 3 | 75 | 27.27 |
| BP00520 | 22 | 23 | 20 | 90.91 | 86.96 |
| BP00526 | 4 | 14 | 4 | 100 | 28.57 |
| BP00562 | 8 | 9 | 8 | 100 | 88.89 |
| BP00922 | 4 | 19 | 4 | 100 | 21.05 |
| BP01002 | 14 | 14 | 14 | 100 | 100 |
| BP01010 | 16 | 16 | 12 | 75 | 75 |
| BP00056 | 24 | 20 | 10 | 41.67 | 50 |
| BP00273 | 16 | 8 | 8 | 50 | 100 |
| BP00339 | 16 | 17 | 16 | 100 | 94.12 |
| BP00431 | 22 | 21 | 18 | 81.82 | 85.71 |
| BP00364 | 4 | 6 | 4 | 100 | 66.67 |
| BP00561 | 8 | 9 | 8 | 100 | 88.89 |
| BP00346 | 22 | 21 | 21 | 95.45 | 100 |
| BP00502 | 2 | 4 | 2 | 100 | 50 |
| **Total** | **442** | **507** | **370** | **83.71** | **72.98** |

The results for each benchmark protein are reported individually.

^a^Sensitivity: TP/(TP+FN), % of all observed transmembrane β-strands predicted correctly by the model.

^b^Precision: TP/(TP+FP), % of all predicted transmembrane β-strands that are correctly predicted.
